# Complete genome sequence of *Microbacterium foliorum* singleton phage Magritte.

**DOI:** 10.17912/micropub.biology.001377

**Published:** 2024-12-06

**Authors:** Elvira Eivazova, Jenna St. Pierre, Miriam Galindo, James Bautista, Annaleisa Matzirakis, Madalyn Falletti, Elynor Fix, Levi Fritsch

**Affiliations:** 1 Columbia State Community College, Columbia, Tennessee, United States

## Abstract

Actinobacteriophage Magritte was isolated from soil in Columbia, TN using *Microbacterium foliorum* as a host. Magritte is a singleton with a siphovirus morphology and a large genome of 133,228 bp encoding 250 predicted genes, including 26 tRNA genes.

**Figure 1. Transmission electron microscopy image of phage Magritte:**
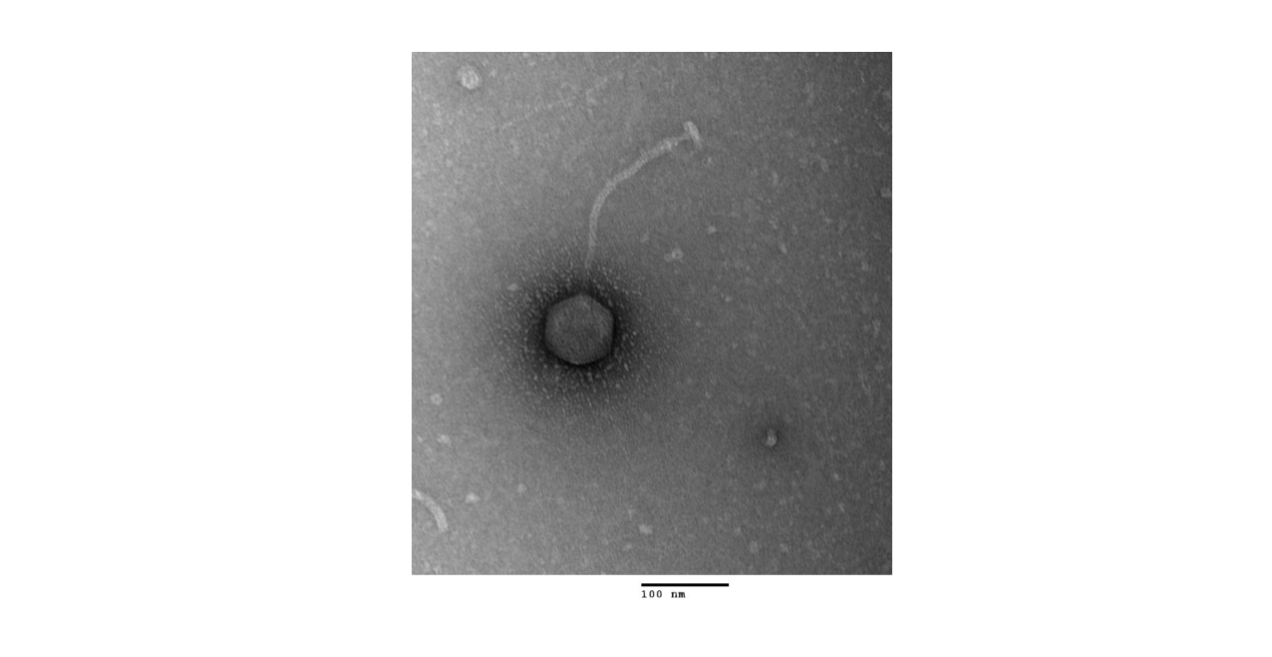
Negative stain (1% uranyl acetate) transmission electron microscopy image of Magritte shows a siphovirus morphology with an icosahedral capsid and a flexible tail. The capsid size and tail length, measured relative to the scale bar of 100 nm, is between 82 to 88 nm in diameter and between 259 to 265 nm, respectively (n=2). A Hitachi H-7650 Transmission Electron Microscope (Tokyo, Japan) was used for imaging with an accelerating voltage of 100 kV.

## Description

Bacteriophages are able to efficiently lyse bacteria and are therefore being developed as potential therapeutics for controlling bacterial growth and infection (Hatfull, 2022) . Phages are genetically diverse and can be clustered based on gene content similarity (GCS) of at least 35% (Hatfull, 2020, Jacobs-Sera, 2020) . Actinobacteriophages with GCS below this threshold are not clustered and are called singletons. Here we present the genome sequence of phage Magritte, a singleton isolated using *Microbacterium foliorum* NRRL B-24224.

Magritte was isolated from a soil sample collected under a sewage pipe in Columbia, TN (35.615 N, 87.1 W) at an ambient temperature of 28°C. The soil sample was washed in PYCa (peptone-yeast extract-calcium) liquid medium, the wash filtered (0.2 μm pore size), and the filtrate inoculated with *M. foliorum* and incubated at 30˚C with shaking. After 72 hours, the culture was filtered and the filtrate plated in top agar supplemented with *M. foliorum* and incubated at 30˚C, resulting in plaques of phage Magritte (Poxleitner, 2018) . Magritte was plaque-purified through 4 rounds of plating and consistently formed small turbid plaques between 2-4 mm in diameter. The concentrated phage sample was placed on a copper grid, negatively stained with uranyl acetate (1%) and imaged using transmission electron microscopy (TEM), revealing a siphovirus morphology as shown in Figure 1 .

Genomic DNA from Magritte was isolated from lysate and purified using the Promega Wizard DNA Clean-Up Kit. A sequencing DNA library was prepared using the NEBNext UltraII Library Kit. The genome was sequenced at the Pittsburgh Bacteriophage Institute on an Illumina MiSeq instrument (v3 reagents) yielding 64,974 single-end 150 bp reads and 65-fold genome coverage. Raw reads were assembled with Newbler v.2.9, and the resulting contig was checked for completeness using Consed v.29. Genomic termini were verified as previously described (Russell, 2018). Magritte has a 133,228 bp long genome with 64.0% GC content and is circularly permuted. Based on gene content similarity below the 35% threshold used to cluster actinobacteriophages, Magritte is classed as a singleton (Russell & Hatfull, 2017; Pope et al., 2017).

The sequence was annotated using DNA Master v.5.23.6 embedded with Glimmer v.3.02 (Delcher, 1999) and GeneMark v.2.5p (Besemer & Borodovsky, 2005) , BLAST (Altschul et al., 1990) against the Actinobacteriophage and NCBI non-redundant databases, HHPred v.3.2 (Söding et al., 2005) against the PDB_mmCIF70, Pfam-v.36, NCBI Conserved Domains databases, Phamerator v.393.0 (Cresawn et al., 2011) , tRNAscanSE v.2.0 (Lowe & Chan, 2016) , Aragorn v.1.2.41 (Laslett & Canback, 2004) , and PECAAN ( http://pecaan.kbrinsgd.org/ ), all using default software settings. Genome annotation revealed 224 putative protein-coding genes, more than 80% of which have no identifiable homologs. The majority of genes that could be assigned putative functions are those involved in virion structure and assembly, including terminase, portal, scaffolding, major capsid, tail terminator, major and minor tail, tail assembly chaperone and tapemeasure proteins. Between genes *85* (HicA-like toxin) and *118* (Ro-like RNA binding protein) are a cluster of 26 tRNA genes. Since no immunity repressor or integrase functions were identified, further experiments are required to confirm the type of phage life cycle.

Data availability. Phage Magritte is available at GenBank with Accession No. OR553905 and Sequence Read Archive (SRA) No. SRX24892099.
